# Note from the Editor: Good Bye and Thank You

**Published:** 2005-12

**Authors:** Thomas J. Goehl

**Affiliations:** Editor-in-Chief, *EHP,* National Institute of Environmental Health Sciences, National Institutes of Health, Department of Health and Human Services

*Environmental Health Perspectives* (*EHP*) is like one of the lighthouses that dot the North Carolina coast; the journal acts as a beacon warning people of potential environmental dangers and, at the same time, welcomes people whose goal is to improve global health. Although we report areas of concern about how our environment can negatively affect us, we also provide information that can give a sense of hope for the improvement of human health. Regardless of the information to be shared, *EHP* tries to provide the balance between voices that sometimes have competing interests.

I will be retiring at the end of December and have been blessed to have a fulfilling career dating back to 1969 that allowed me to experience industrial, academic, and governmental service. Along the way I have had the honor to work with many wonderful people. However, none of my experiences has been more fulfilling than my time at NIEHS during which I served with the National Toxicology Program and now with *EHP*. The talent, energy, tenacity, and altruism of these wonderful people are beyond compare.

NIEHS has been the benevolent sponsor of *EHP* since its inception over 30 years ago. NIEHS is an exceptional institution with exceptional people, who are at the forefront of research in environmental health issues. During this time *EHP* has given a voice to the institute and to the field of environmental health.

Although the future sponsorship of *EHP* is uncertain, the journal will continue in its mission of serving “as a forum for the discussion of the interrelationships between the environment and human health by publishing in a balanced and objective manner the best peer-reviewed research and most current and credible news of the field.”

My immediate plans are to go on extended camping trips across the United States with my wife, Marilyn (who is *not* just another pretty face!). I hope to use this time to contemplate new ways to contribute to scientific capacity building and information dissemination in the developing world, which has been my passion at *EHP*. I am very grateful for, and will always remember, the support and dedication of the *EHP* staff and the NIEHS administration during my tenure.

